# The Microbiome–Mitochondria–Extracellular Vesicle Axis in HPV Persistence and Cervical Carcinogenesis

**DOI:** 10.3390/genes17060655

**Published:** 2026-06-01

**Authors:** Efthalia Moustakli, Stylianos Makrydimas, Emmanouil D. Oikonomou, Agni Nakou, Eleni Albani, Nektaria Zagorianakou

**Affiliations:** 1 Department of Nursing, School of Health Sciences University of Ioannina, 4th Kilometer National Highway Street Ioannina-Athens, 45500 Ioannina, Greece; agni-nk@hotmail.com; 2 Medical School, Aristotle University of Thessaloniki, 54124 Thessaloniki, Greece; smakrydimas@gmail.com; 3 Human Computer Interaction Laboratory, Department of Informatics and Telecommunications, University of Ioannina, Kostakioi, 47150 Arta, Greece; e.oikonomou@uoi.gr; 4 Laboratory of Child Care and Family Resilience, Department of Nursing, University of Patras, 26334 Patras, Greece; ealbani@upatras.gr; 5 Scientific Laboratory for Innovative Technologies in Internal Medicine, Preventive Medicine and Overall Care, Department of Nursing, School of Health Sciences, University of Ioannina, 45500 Ioannina, Greece

**Keywords:** vaginal microbiome, gut microbiome, oxidative stress, mitochondrial dysfunction, innate immunity, viral immune evasion, vaccination, disease elimination

## Abstract

Persistence of human papillomavirus (HPV) infection leading to cervical carcinogenesis can be attributed to the action of high-risk HPVs, but there are still some unclear factors involved in the mechanisms of either viral clearance or persistence. Although many infections may be self-limiting and cleared successfully by the immune response of the infected individuals, other infections result in persistent HPV infection. Recent studies indicate that microbiota in the gut and cervicovaginal tract modulate host immune status, mucosal inflammation, and epithelial barrier integrity. All these factors determine susceptibility to persistent infection. Inflammation, overproduction of reactive oxygen species (ROS), genomic instability, and impaired antiviral transcription pathways are associated with dysbiosis. In parallel, redox imbalance contributes to mitochondrial dysfunction, impairing mitochondrial antiviral signaling (MAVS)-dependent interferon responses and attenuating induction of interferon-stimulated genes. Additionally, extracellular vesicles (EVs) further promote immune evasion, metabolic programming, and epigenetic regulation by facilitating the intercellular exchange of viral constituents, microRNAs, and signaling molecules. Through this interconnected network of mechanisms, microbial dysbiosis, mitochondrial disruption, and EV signaling collectively shape a niche conducive to persistence. Unlike previous reviews that primarily examine microbiome alterations, oxidative stress (OS), mitochondrial dysfunction, extracellular vesicles, or immune responses as separate processes, this review integrates clinical and omics findings into a systems-based conceptual framework of HPV persistence. By emphasizing the potential interactions among these interconnected biological systems, we aim to identify points of biological convergence, generate mechanistic hypotheses, and highlight opportunities for future biomarker development and therapeutic intervention.

## 1. Introduction

Cervical cancer is a classic example of viral oncogenesis and continues to be a leading cause of cancer morbidity and mortality in females globally. A persistent infection with a specific type of human papillomavirus (HPV) is a causal factor for cervical carcinoma [1,2,3]. Although infections from HPV are very common, especially just after sexual debut, the majority of infections will be temporary and will disappear in about a year. High-grade cervical intraepithelial neoplasia (CIN) and cancer can occur only when the infection persists rather than at the time of acquisition of the virus. Only a few viruses succeed in resisting immunoregulation and continue their existence [4,5].

The factors at the molecular level that discriminate between viral clearance and persistent infection have not been fully elucidated. Examples of classic cofactors include smoking, hormonal influences, reproduction, coinfection, and immunosuppression. These may influence disease outcomes but do not fully explain inter-individual variability [6]. At the cellular level, viral clearance depends on the coordinated activation of innate immunity, mitochondrial antiviral signaling (MAVS)-mediated interferon production, interferon-stimulated genes (ISGs), antigen presentation, and cytotoxic T cells [7]. High-risk HPV types have adapted means by which they suppress interferon pathways, apoptotic responses, and inflammatory responses, thus allowing them to persist within the basal epithelium [8,9,10]. However, viral immune evasion alone does not fully account for the stability and heterogeneity of persistent infection.

Increasing data indicate that the cervicovaginal environment represents an intricate ecosystem involving interactions between indigenous microbiomes, immune monitoring, hormonal influence, and epithelial biology [11,12,13,14]. The presence of a low-diversity microbiome enriched in Lactobacillus species indicates a healthy reproductive system, whereas inflammatory conditions and poor gynecological health are associated with Lactobacillus depletion and increased colonization by diverse anaerobic bacteria, a state termed dysbiosis [15,16]. Cross-sectional and longitudinal studies indicate that dysbiosis is associated with increased HPV prevalence, reduced viral clearance, and a higher incidence of CIN, suggesting that microbiome composition significantly influences viral pathogenesis [17]. From a mechanistic standpoint, dysbiosis can compromise epithelial barrier function, modulate mucosal immune tone, and drive chronic inflammation accompanied by elevated reactive oxygen species (ROS).

In addition to direct genotoxic effects, sustained redox dysregulation has wider regulatory implications. Elevated levels of ROS may lead to mitochondrial dysfunction, disruption of MAVS-mediated interferon signaling, and reduced transcription of antiviral genes [18,19]. However, oxidative stress (OS) and metabolic rewiring may lead to epigenetic modifications, such as abnormal DNA methylation and chromatin modification, which help maintain transcriptional programs that promote virus persistence [20].

Moreover, cell-to-cell signaling enhances these mechanisms. Extracellular vesicles (EVs), which are secreted by cells undergoing infection or cellular stress, carry viral components, microRNAs (miRNAs), and other molecules that regulate gene expression, metabolism, and immunity in target cells [21,22]. By spreading immunoregulatory and epigenetic signals through EVs, local disruptions can spread to neighboring epithelial and immune cells, thereby contributing to viral persistence and creating an environment conducive to tumor formation [23,24].

While the HPV vaccine has greatly minimized the incidence of vaccine strains and severe pre-cancers, HPV persistence is still a major issue among those who do not receive the vaccine, have had previous exposure, or lack access to preventive healthcare. An improved understanding of the persistence process is essential for advancing risk stratification and identifying novel biomarkers and therapeutic targets [25,26]. Importantly, although increasing evidence supports interactions among cervicovaginal dysbiosis, OS, mitochondrial signaling, extracellular vesicle biology, and host antiviral responses, the strength of evidence varies across pathway components. In this review, we distinguish findings derived directly from HPV infection and cervical carcinogenesis from mechanistic insights originating from broader viral, inflammatory, mitochondrial, and oncologic research. Accordingly, the proposed microbiome–mitochondria–extracellular vesicle axis should be interpreted as an integrative conceptual framework that generates mechanistic hypotheses rather than representing a fully established causal pathway.

In this review, we present a systems-level approach that involves microbial dysbiosis, redox dysregulation, dysregulated mitochondrial immune signaling pathways, epigenetics, and exosomal signaling as factors that can influence viral clearance or persistence. This review explores how ecological disruption drives sustained gene regulatory dysfunction, ultimately contributing to cervical cancer.

## 2. Literature Search Strategy

Literature searches were conducted using a structured approach to identify studies on HPV persistence and its relationships with the microbiota, mitochondria, OS, epigenetics, and extracellular vesicles.

Electronic databases, including PubMed, Scopus, and Web of Science, were systematically searched for articles published up to January 2026. Search terms were combined using Boolean operators and included: “HPV persistence”, “cervical cancer”, “vaginal microbiome”, “cervicovaginal microbiota”, “dysbiosis”, “oxidative stress”, “reactive oxygen species”, “mitochondria”, “mitochondrial dysfunction”, “MAVS”, “interferon signaling”, “epigenetics”, “DNA methylation”, “extracellular vesicles”, “exosomes”, and “microRNA”.

Priority was given to longitudinal cohort and mechanistic studies, as well as influential reviews, to elucidate molecular mechanisms and host–microenvironment interactions. Papers beyond those found by literature searches were identified manually from the references of relevant papers.

Only English-language studies were included. No time restrictions were applied, although recent studies with sound methodology and translational relevance were preferred.

A literature review was conducted in order to synthesize an understanding of the relationship between microbial dysbiosis, redox disturbance, mitochondrial dysfunction, and communication through extracellular vesicles in HPV-induced cervical carcinogenesis.

## 3. Natural History of HPV Infection and Determinants of Persistence

HPV infection ranks among the most common sexually transmitted viral infections. Its incidence peaks shortly after an individual’s first sexual intercourse; however, in most cases, the infection becomes undetectable within one to two years due to immune-mediated clearance, although this does not confer sterilizing immunity [27,28]. On the contrary, persistently detecting the same type, especially HPV16, constitutes the best predictor of developing CIN/carcinoma [29].

Persistence is now recognized as resulting from poor antiviral surveillance rather than solely from infection [30]. Host-based cofactors such as smoking, hormonal status, reproductive aspects, co-infection, and immunosuppression affect risk but do not fully account for outcome variation between individuals [31]. In immunocompetent individuals, subtle variations in immune detection, interferon responses, antigen presentation efficiency, and cytotoxic lymphocyte activity may critically influence viral fate [32,33,34].

Oncoproteins of high-risk HPVs have been found to disrupt the antiviral defenses of the body’s cells by blocking apoptosis, preventing interferon production, and reducing antigen presentation [35]. However, this alone cannot account for the persistence of the infection. The periodic presence and absence of the virus indicate that there is a balance between viral gene expression and host immunity [36,37,38].

Evidence is accumulating to suggest that greater shifts in the immune-metabolic environment within the epithelium are necessary for persistence. Disruptions in redox balance, mitochondrial communication, and the regulation of antiviral genes may create conditions that allow infected basal keratinocytes to survive [39,40,41]. These effects may be further amplified by inflammatory signals that progressively render the tissue permissive to viral persistence.

In summary, these data indicate that persistent infection arises from a failure of coordination among antiviral responses, host immune variation, metabolic disturbances, and gene regulatory dysfunction. This enables consideration of interactions among extracellular vesicles, OS, mitochondrial dysfunction, microbiota dysbiosis, and epigenetic modifications in the context of viral infection.

## 4. Cervicovaginal Microbiome and HPV Persistence

The cervicovaginal microenvironment contains a diverse community of microbes whose activity contributes greatly to the maintenance of mucosal homeostasis [42,43]. Culture-independent DNA sequencing experiments have revealed that, in most cases, healthy women of reproductive age have a vaginal microbiome characterized by low-diversity bacterial communities dominated by Lactobacillus species [44,45]. In contrast, increased microbial diversity and reduced Lactobacillus abundance are often associated with inflammation and adverse reproductive conditions [46].

The vaginal microbiome is commonly classified into community state types (CSTs) based on its composition [44]. CSTs enriched in Lactobacillus species, particularly Lactobacillus crispatus, are associated with low vaginal pH, reduced inflammation, and greater mucosal integrity [47,48]. However, CSTs enriched in anaerobes such as *Gardnerella*, *Prevotella*, *Sneathia*, and *Atopobium* are associated with dysbiosis and inflammation [49,50].

It has been established that the natural history of HPV infection is very much associated with this particular microbial composition. A study comparing women with *Lactobacillus*-dominated microbiota to those without found similar associations with HPV infection [51,52,53].

Although causal relationships remain to be fully established, multiple studies across diverse populations provide converging evidence for an association between microbial community structure and HPV persistence [54]. However, much of the available evidence derives from observational and cross-sectional studies, limiting causal inference and leaving the possibility of confounding factors and bidirectional host–microbiome interactions. Anaerobic, high-diversity microbiomes are more frequently associated with persistence and progression, whereas *Lactobacillus*-dominated microbiomes are linked to viral clearance and lesion regression.

A central mechanism linking microbial composition to viral outcomes is the maintenance of epithelial barrier integrity. Through the production of lactic acid, *Lactobacillus* species contribute to an acidic vaginal environment that limits pathogen colonization and supports mucosal homeostasis. Depletion of lactobacilli may therefore impair barrier function, facilitating viral access to basal keratinocytes and increasing the likelihood of persistent infection [30].

Evidence from invasive cervical cancer further supports a progressive shift toward diverse, *Lactobacillus*-depleted microbial communities accompanied by distinct cytokine profiles, extending observations from precancerous stages. These findings suggest that microbiome-associated inflammation contributes to both viral persistence and the establishment of a tumor-permissive microenvironment. In addition, enzymes produced by dysbiotic communities can degrade mucus and extracellular matrix components, further compromising tissue integrity and increasing susceptibility to viral infection [30,31].

Temporal variability in microbial composition may represent an additional contributing factor. Fluctuations between *Lactobacillus*-dominated and anaerobe-rich states may lead to repeated alterations in local immunity and epithelial vulnerability, potentially explaining the episodic detection of HPV observed in longitudinal studies.

Importantly, not all *Lactobacillus* species confer equal protection. Emerging evidence suggests that *Lactobacillus crispatus* is associated with normal cervical cytology and effective viral clearance, whereas Lactobacillus iners may represent a more transitional or less protective state [33].

In summary, the cervicovaginal microbiota act as key regulators of the epithelial microenvironment, influencing antiviral defense through coordinated effects on barrier integrity, inflammation, and cellular stress responses. An overview of these associations and underlying mechanisms is provided in Table 1.

## 5. Microbiome-Driven Inflammation and OS in HPV Persistence

Evidence supporting interactions among cervicovaginal dysbiosis, OS, mitochondrial signaling, and antiviral immunity varies considerably in strength and biological context. While associations between microbial composition and HPV persistence have been reported, many mechanistic links discussed in this section derive from the broader literature on inflammation, antiviral immunity, and carcinogenesis rather than direct HPV-specific experimental evidence. Therefore, the pathways described below should be interpreted as an integrative mechanistic framework rather than a fully established causal sequence.

Microbial inflammation within the cervicovaginal mucosa has been linked to imbalance of the cervicovaginal microflora. Inflammatory markers, including proinflammatory cytokines, chemokines, and immune cells, are elevated in patients with anaerobe-dominated microbiota compared with those with *Lactobacillus*-dominated microflora [14,59]. While inflammation plays a crucial role in host defense, continuous or abnormal inflammation signaling can result in virus persistence.

Chronic inflammation leads to sustained production of ROS and RNS, resulting in a genotoxic and immunomodulatory redox environment. This includes damage to DNA, altered regulation of the cell cycle, as well as mutagenic processes that favor carcinogenesis [57,61]. Regarding HPV infection, OS plays a role in the process of viral genomic instability and integration, which is directly related to malignancy progression [58,62].

Disruption of normal microbial flora may further contribute to oxidative imbalance. Anaerobic bacteria produce metabolites capable of inducing inflammatory responses and increasing oxidative burden within epithelial tissues. Although these mechanisms have been associated with conditions permissive to viral persistence, direct evidence demonstrating that microbiome-induced OS promotes HPV persistence remains limited [63,64,65].

Although this review primarily focuses on the cervicovaginal microbiome, emerging evidence suggests that gut microbial composition may also indirectly influence HPV persistence through systemic immune and metabolic regulation. Gut-derived inflammatory mediators, immune cell modulation, and circulating microbial metabolites, including short-chain fatty acids, have been proposed to affect mucosal immune responses beyond the intestinal environment. Through these mechanisms, a potential gut-cervical axis may contribute to host antiviral competence and cervical microenvironment regulation; however, direct evidence linking gut microbiota to persistent HPV infection remains limited.

Mechanistically, anaerobe-rich dysbiotic communities may promote epithelial vulnerability through the production of sialidases, mucinases, and proteolytic enzymes capable of degrading mucin-associated glycans and extracellular matrix components, thereby weakening the mucus barrier and increasing epithelial permeability. In addition, enzymatic activity associated with genera such as *Prevotella* and *Gardnerella* may generate pro-inflammatory metabolites and microbial products, including short-chain fatty acid derivatives and mucin degradation products, which can activate epithelial and innate immune signaling pathways. These processes may enhance cytokine production, neutrophil recruitment, local ROS/RNS generation, and altered immune responsiveness. Although such mechanisms provide biological plausibility for a role in HPV persistence, direct causal evidence in HPV-specific models remains incomplete.

The virus HPV contributes to an even higher level of pro-oxidant state in epithelial tissues. HPV-encoded oncogenes, primarily E6 and E7, inhibit host cell antioxidant defenses, leading to genomic instability and the accumulation of reactive species in infected tissues [58,62,66]. Collectively, available evidence suggests that microbial dysbiosis and HPV-associated oxidative pathways may interact to sustain inflammatory and redox imbalance. However, the extent to which these processes constitute a coordinated positive-feedback mechanism in HPV persistence remains incompletely established [63,67].

Beyond genotoxicity, redox imbalance modulates immune processes. Specifically, OS may cause defective antigen presentation, T-cell activation, and cytokine signaling. These factors impair the body’s ability to detect and eliminate pathogens within infected cells [68,69]. Supporting this theory, studies have shown the presence of biomarkers of DNA oxidative damage at elevated levels in women suffering from cervical precancer compared to the healthy group.

Overall, these findings support the hypothesis that redox imbalance may represent one potential interface between microbial alterations and antiviral immune regulation; however, causal relationships remain to be experimentally validated [64,65,70]. Emerging evidence suggests that OS may influence mitochondrial homeostasis and downstream antiviral pathways, although the relevance of these mechanisms to persistent HPV infection remains incompletely understood [62]. These interactions are discussed further in the following section [71,72,73].

## 6. Mitochondrial Dysfunction and Failure of Antiviral Surveillance

Although mitochondrial regulation of antiviral immunity is increasingly recognized, the degree of evidence varies across biological contexts. In this section, direct observations from HPV biology are discussed alongside mechanistic insights derived from broader antiviral, inflammatory, and cancer research.

There is now an appreciation of mitochondria as key regulators in antiviral innate immunity. In addition to its conventional role in cellular respiration, the mitochondria serve as a signaling platform that regulates interferon response, apoptosis, and inflammation [74,75]. MAVS is a central mediator that integrates signals from upstream pattern recognition receptors and activates type I interferon signaling, resulting in the induction of interferon-stimulated genes essential for viral clearance [76,77].

Consequently, mitochondrial integrity appears to contribute to effective antiviral responses and may influence the clearance of virus-infected cells. Interference with mitochondrial dynamics, mitochondrial membrane potential, or cellular metabolism significantly reduces the ability to respond to viruses. Impaired MAVS has been associated with reduced interferon responses and persistent infection across multiple viral systems; however, direct evidence supporting this mechanism in HPV persistence remains limited [78,79,80].

Redox imbalance may hinder MAVS at several molecular levels. The architecture of the outer mitochondrial membrane, where MAVS forms signaling aggregates following upstream RIG-I-like receptor activation, can be disrupted by excess mitochondrial ROS and mitochondrial membrane depolarization. This might restrict phosphorylation and nuclear translocation of IRF3 and IRF7, attenuate downstream TBK1/IKKε activation, and decrease recruitment of TRAF3 and TRAF6 adaptor proteins. These changes could reduce the activation of interferon-stimulated genes and decrease IFN-β signaling, according to experimental research in antiviral immunity. Nevertheless, there is still little direct evidence of this sequence in cervical epithelial cells infected with HPV.

Mitochondrial OS is a major contributor to mitochondrial dysfunction. Mitochondrial DNA can be damaged by excessive ROS generation, which may result in the disruption of the electron transport chain and the development of abnormal signal states [81,82]. These alterations may favor cellular survival pathways over apoptotic responses and have been proposed to contribute to persistence-promoting conditions.

Evidence suggests that high-risk HPV may modulate mitochondrial function in ways that support cellular adaptation and persistence. High-risk HPV oncoproteins, such as E6 and E7, disrupt mitochondrial metabolic activity, inhibit apoptosis, and reprogram cellular energy metabolism to promote proliferation [41,83]. These alterations have been proposed to influence innate immune signaling and favor survival of infected keratinocytes.

Immune cell behavior is similarly dependent on mitochondrial function. The differentiation and functional activities of T lymphocytes, macrophages, and dendritic cells, which are crucial to the clearance of cells harboring HPV infections, are regulated by metabolic reprogramming [84,85,86]. Therefore, mitochondrial dysfunction may influence immune cell behavior and antiviral responses, although the extent to which these mechanisms contribute directly to HPV persistence remains incompletely defined [83].

New studies also reveal the role of mitochondrial defects in establishing an immunosuppressive environment. Reduced interferon activity, impaired antigen processing, and anti-apoptotic mechanisms promote survival and immune evasion of HPV-infected and transformed cells [41,87,88]. Cervicovaginal dysbiosis, chronic inflammatory signaling, and microbial metabolites have been associated with mitochondrial stress and altered immune responses; however, whether these processes form a unified pathogenic cycle in HPV persistence remains to be experimentally established [89].

Collectively, these observations support the hypothesis that mitochondria may represent an important interface connecting metabolic stress, redox regulation, and antiviral signaling. However, current evidence remains heterogeneous and derives from both HPV-specific and broader mechanistic studies. Additional experimental work is needed to determine whether mitochondrial dysfunction and MAVS-associated signaling constitute a central driver of persistent HPV infection. The principal redox-driven mitochondrial alterations implicated in HPV persistence are summarized in Table 2.

## 7. EVs as Amplifiers of HPV Persistence and Microenvironmental Reprogramming

The ensuing immunometabolic state is not limited to individual cells but can spread across the tissue milieu if mitochondrial and redox homeostasis are upset. EVs have become important mediators of this process, allowing stressed or infected epithelial cells to spread metabolic and immunoregulatory signals across the cervicovaginal niche [91,92,93].

Nearly all cell types generate EVs, which are heterogeneous lipid-bilayer particles that transport a variety of molecular cargo, such as proteins, lipids, DNA, mRNA, and non-coding RNAs. By transferring this cargo, EVs can prolong the effects of localized infection by altering recipient cells’ gene regulation systems, cellular metabolism, and immune surveillance [24,94].

### 7.1. EV Biogenesis and Functional Heterogeneity

Different biogenetic routes give birth to EVs, which have an impact on their composition and functionality. Microvesicles emerge directly from the plasma membrane, whereas exosomes originate from multivesicular bodies and are liberated during membrane fusion. This variability promotes functional diversity but complicates standardization. EV profiles in cancer can actively modulate the tumor microenvironment and reflect cellular stress and oncogenic signaling states [95,96,97].

### 7.2. EVs in Viral Infection: Immune Evasion and Signal Dissemination

In viral infections, EVs represent an additional layer of host–pathogen interaction. Vesicles derived from infected cells can carry viral components and host-derived immunoregulatory molecules, thereby affecting the susceptibility of recipient cells to infection and immune activation thresholds [98,99,100]. Vesicle-mediated transfer provides a stealth mechanism for dispersing viral and regulatory signals throughout tissues because EV membranes protect their contents from immune detection [101]. These principles are increasingly recognized in HPV-associated disease.

### 7.3. HPV-Associated EVs: Cargo, Immune Modulation, and Oncogenic Conditioning

EVs help create a microenvironment that promotes viral persistence and oncogenic development in HPV-associated cervical conditions [102,103,104,105]. It has been revealed that EV cargo in HPV-associated illness reflects the activation of viral oncoproteins and modified host signaling pathways. According to experimental research, HPV-positive cells may produce EVs that are rich in regulatory microRNAs linked to E6/E7 expression as well as immune-modulatory proteins that can affect cytokine signaling, epithelial–immune communication, and antiviral response. Although there is yet no direct mechanistic evidence, these data point to a possible role for HPV-derived EV cargo in influencing persistence-associated microenvironmental alterations.

Vesicle contents can affect immunological recognition, angiogenesis, migration, and cellular proliferation, all of which can support viral maintenance. Mechanistically, EV cargo may reshape recipient-cell signaling through the transfer of regulatory microRNAs, mitochondrial metabolites, inflammatory mediators, and proteins involved in immune regulation. Internalization of EVs by epithelial and immune cells can alter transcriptional programs associated with interferon responsiveness, antigen presentation, and cellular metabolism. Experimental evidence further suggests that EV-mediated signaling may promote immune tolerance through suppression of antiviral pathways and induction of persistence-associated cellular phenotypes. EV-mediated signaling, therefore, contributes to coordinated microenvironmental reprogramming by extending the effects of HPV beyond directly infected cells [22,23,106].

### 7.4. EV miRNAs and Non-Coding RNAs in Long-Range Gene Regulation

One important way that EVs affect persistence is through the transfer of regulatory RNAs. In recipient cells, EV-associated microRNAs and other non-coding RNAs can alter gene expression patterns, such as activating survival pathways and suppressing interferon signaling [24,99,107]. Exosomal microRNAs that reflect the transcriptional and immunological status of the tumor microenvironment are both functional regulators and potential biomarkers in cervical cancer [108].

### 7.5. The EV–Mitochondria Axis: Metabolic and Redox Reprogramming

Additionally, EVs may directly affect the mitochondrial activity of receiving cells. Antiviral signaling pathways may be weakened, redox imbalance can be reinforced, and mitochondrial dynamics can be changed by the delivery of metabolites, enzymes, and regulatory RNAs [109,110,111]. At the cellular level, EV-mediated transfer of regulatory RNAs and metabolic effectors may influence mitochondrial membrane potential, mitochondrial dynamics, and ROS generation. These changes may interfere with MAVS platform integrity and reduce activation of downstream TBK1–IRF signaling cascades, ultimately decreasing transcription of interferon-responsive genes. Such mechanisms provide a plausible explanation for how localized metabolic stress may propagate across neighboring non-infected cells. This EV–mitochondria axis supports a theory that EVs spread mitochondrial dysfunction and compromised interferon responses across the tissue milieu by establishing a mechanistic connection between extracellular communication and intracellular antiviral capability [111,112,113].

### 7.6. EV-Mediated Immune Regulation in Cancer and Infection

Persistence is mostly dependent on EV-mediated immune regulation. Immunosuppressive cell populations can proliferate, cytotoxic T-cell activity can be decreased, and antigen presentation can be changed by vesicles. These results align with the immunological landscape seen in chronic HPV infection, where inflammatory signaling persists but antiviral responses are diminished [114,115,116].

### 7.7. Clinical Translation: EVs as Biomarkers and Liquid Biopsy Tools

EVs are interesting candidates for non-invasive biomarkers because they are easily identified in biofluids and retain molecular information representative of both immune and epithelial compartments [117]. Proteins and nucleic acids carried by EVs may enable dynamic monitoring of therapy response, disease progression, and the risk of viral persistence. The promise of EV-based “liquid biopsy” techniques in HPV-associated illness is highlighted by recent research [108].

**Figure 1:**
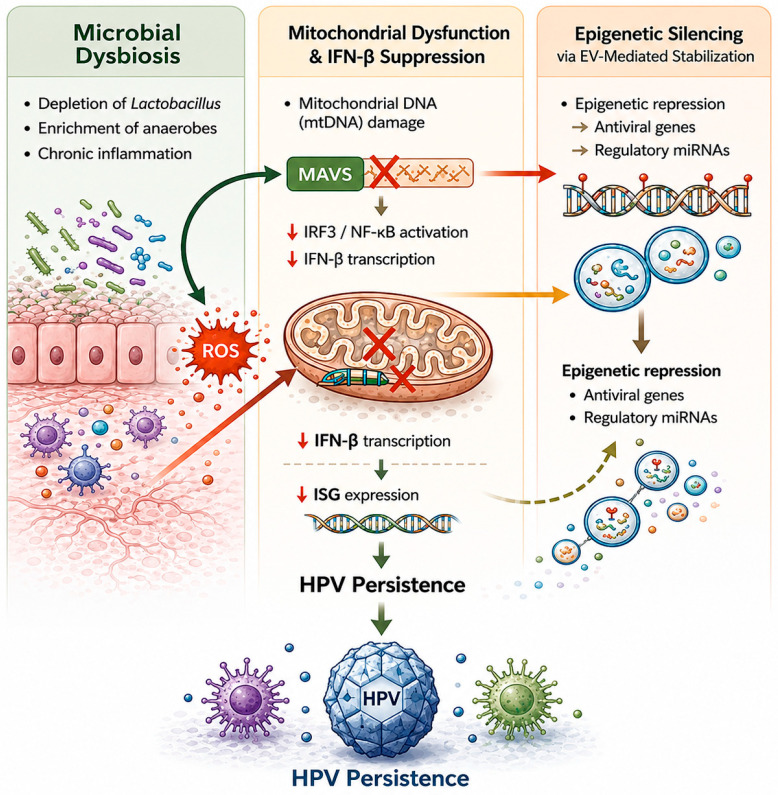
Integrated mechanisms underlying HPV persistence. Microbial dysbiosis, marked by Lactobacillus depletion and anaerobic enrichment, promotes inflammation and reactive oxygen species (ROS) production, leading to mitochondrial damage and impaired MAVS-mediated antiviral signaling. This reduces IFN-β transcription and interferon-stimulated gene (ISG) expression, weakening host defense. Concurrently, extracellular vesicles (EVs) facilitate epigenetic repression of antiviral genes, further suppressing immune responses. Together, these processes create a cellular environment conducive to persistent HPV infection.

Collectively, EVs can be conceptualized as signal amplifiers that propagate immune-modulatory, metabolic, and gene-regulatory programs across the cervicovaginal microenvironment. EV-mediated communication may be crucial in maintaining HPV survival by connecting dysbiosis-driven inflammation and OS to mitochondrial dysfunction and compromised antiviral signaling [41,105]. The interactions between microbial imbalance, OS, mitochondrial failure, and EV signaling are shown in Figure 1.

## 8. Integrated Microbiome–Mitochondria–EV Crosstalk in HPV Persistence

According to a unifying interpretation of the available data, HPV persistence reflects a systems-level shift in the cervicovaginal ecosystem from a clearance-permissive state to one that stabilizes viral maintenance. This shift results from convergent changes in microbial composition, inflammatory tone, redox balance, mitochondrial competence, and intercellular communication networks rather than being solely determined by viral exposure [11,17]. A summary of the principal system-level alterations and their mechanistic contributions to antiviral dysfunction and HPV persistence is presented in Table 3.

### 8.1. From Microbial Community Structure to Mucosal Immune Tone

However, there are variations in both the taxonomy and functions of the cervicovaginal microbiome, which impact mucosal immune responses. Anaerobe-rich, high-diversity microbiomes are associated with pro-inflammatory profiles and increased detection of persistent hrHPV, whereas lactobacilli-dominated communities are linked to epithelial health and anti-inflammatory states [14,54,55]. Multiple longitudinal cohort studies support the hypothesis that microbial community structure influences clinical endpoints rather than merely reflecting them, with Lactobacillus depletion strongly associated with persistence and progression to higher-grade hrHPV disease [54]. Dysbiosis creates a chronic inflammatory baseline state that induces ongoing tissue stress without eliminating the virus.

**Table 3:** Multiscale interactions among the microbiome, redox signaling, extracellular vesicles, and antiviral defense in HPV persistence.

System Level	Key Alterations	Mechanistic Consequences	Impact on Antiviral Defense	Relevance to HPV Persistence
Microbiome composition [14,54,55]	Lactobacillus depletion; anaerobe enrichment; increased diversity	Chronic inflammation; epithelial barrier disruption	Altered mucosal immunity and cytokine profiles	Promotes hrHPV persistence and progression
Inflammatory and redox state [70,81]	Elevated ROS; chronic inflammatory signaling	Oxidative microenvironment; epithelial stress	Impaired immune coordination	Supports persistence-promoting tissue remodeling
EV communication [17,70]	Transfer of regulatory RNAs, proteins, metabolic signals	Spread of immunosuppressive and metabolic reprogramming signals	Amplified suppression of local immune responses	Reinforces persistence niche across tissue
Tissue microenvironment [18,79]	Immune modulation; metabolic reprogramming; epithelial stress	Crosstalk between epithelial, immune, and stromal cells	Reduced antiviral competence at tissue level	Facilitates immune evasion and persistence
Disease progression [60,90]	Viral genome instability and integration	Oncogenic transformation pathways activation	Loss of immune surveillance	Drives progression to cervical cancer
Clinical implications [79,118]	Composite biomarkers (microbiome profiles, EV cargo, OS markers)	Improved risk stratification and targeted interventions	Potential restoration of antiviral responses	Supports elimination strategies beyond vaccination

### 8.2. OS Linking Dysbiosis to Mitochondrial Dysfunction

Redox imbalance is an important link between microbial signaling and antiviral responses within the host. Increased ROS generation during inflammation caused by microbial dysbiosis leads to impaired mitochondrial biogenesis, damage to nuclear and mitochondrial DNA, and mitochondrial membrane depolarization [70,81]. This situation is especially significant, as mitochondria play a key role in antiviral signaling.

Mitochondrial antiviral signaling occurs via MAVS-dependent pathways that activate interferon responses and antiviral genes. As noted above, interferon signaling is impaired when mitochondrial function is disrupted, creating conditions that favor viral persistence [119,120]. Given mitochondrial dysfunction, OS might be considered both a genotoxic agent and a source of insufficient antiviral signaling.

### 8.3. EVs in the HPV Persistence Microenvironment

Localized disruptions can spread across the tissue microenvironment through the use of extracellular vesicles. By transferring regulatory RNAs, proteins, and metabolic mediators, EVs allow stressed or infected cells to affect nearby immunological, stromal, and epithelial cells [24,121]. This is especially important in lesions linked to HPV, as broad microenvironmental reprogramming can be caused by spatially limited infection.

Through this mechanism, EVs act as network multipliers, spreading immunosuppressive and metabolically altered states across the cervicovaginal niche. This reinforces mitochondrial dysfunction and attenuates antiviral competence [18]. Beyond passive signal dissemination, EV-mediated communication may function as a feed-forward regulatory mechanism whereby oxidative and inflammatory signals generated within infected regions induce secondary transcriptional and metabolic changes in adjacent cells. Through coordinated modulation of immune signaling, mitochondrial activity, and epithelial stress responses, EVs may contribute to stabilization of a persistence-supporting tissue state. However, differences in isolation and characterisation techniques may affect the interpretation of functional effects, making methodological rigor crucial in EV research [118,122].

### 8.4. A Stepwise Model for HPV Persistence and Progression

HPV persistence may represent a progressive systems-level shift rather than a single-pathway event. This is preceded by alterations in the microbiome, characterized by depletion of lactobacilli and enrichment of anaerobes. This leads to enhanced inflammation as well as a defective epithelial barrier, which then leads to enhanced tissue stress, deranged redox balance, and increased generation of ROS [123,124]. Increased OS and mitochondrial dysfunction impair MAVS-dependent interferon signaling and apoptosis in infected cells [125].

Mitochondrial dysfunction weakens antiviral defense, allowing infected keratinocytes to survive while harboring viral DNA in their nuclei. At the molecular level, impaired MAVS may reduce recruitment of downstream signaling adaptors and limit interferon induction, while EV-mediated redistribution of regulatory cargo amplifies these effects across the local tissue environment. This sequential interaction provides a mechanistic framework linking dysbiosis-induced inflammation to sustained antiviral suppression and eventual viral persistence. Simultaneously, extracellular vesicles amplify persistence-promoting environments by disseminating immunomodulatory and metabolic signals throughout the tissue milieu, thereby promoting immune escape and metabolic programming [18,79].

This will allow for viral genome instability and the integration of viral genomes into chromosomes, resulting in carcinogenesis. Overall, this concept emphasizes that HPV persistence results from coordinated disruption across microbial, immunological, metabolic, and gene regulatory systems, helping to explain why single-axis therapies frequently have limited efficacy [60,90].

### 8.5. From Persistence Biology to HPV Elimination Strategies

However, persistent infections remain prevalent in unvaccinated and immunocompromised communities despite the success of vaccine interventions in reducing the prevalence of high-risk HPV infections and lesions. This calls for supplementary measures to be put in place beyond the prevention of infections, as illustrated in the World Health Organization’s eradication strategy (90-70-90) [126,127,128].

Therefore, any effort to eradicate HPV will greatly benefit from a mechanistic understanding of HPV persistence [129,130]. The identification of biomarkers of EV-mediated communication, mitochondrial biology, OS, and microbiota may be useful in risk stratification and therapy targeting. Particularly in under-vaccinated populations, this strategy may promote viral clearance and slow disease progression [118,129].

## 9. Biomarkers, Risk Stratification and Therapeutic Opportunities

The identification of women whose high-risk HPV infection will continue and worsen is a major obstacle in the prevention of cervical cancer. Although molecular HPV testing has significantly improved screening sensitivity, viral DNA detection is insufficient to distinguish between transient infection and progressive disease [130,131].

The greater protective benefit of HPV-based screening over cytology has been shown by large randomized trials and long-term follow-up studies [132,133,134]. However, the high frequency of temporary infections calls for efficient triage methods that can identify those who are actually at risk of cancer and CIN2+ [131,135]. As a result, biomarkers that reflect host cellular changes and viral oncogene activity, rather than viral presence alone, are becoming increasingly important.

The combination detection of p16INK4a and Ki-67 is one of the most proven methods. Co-expression of these markers within the same cell enhances specificity for detecting precancerous lesions in HPV-positive women and reflects HPV oncogene-induced dysregulation of the cell cycle [136,137,138,139]. Dual-stain testing is a key example of the shift toward biologically guided triage and is increasingly incorporated into risk-based screening algorithms.

Another quickly developing field is epigenetic biomarkers. Persistent infection and lesion severity have been linked to aberrant DNA methylation in host genes involved in immune control, differentiation, and tumor suppression. Methylation panels identified using both targeted and genome-wide techniques have shown promising diagnostic performance for detecting high-grade disease [67]. Crucially, these changes could represent the combined impacts of microenvironmental stress, chronic inflammation, and viral persistence.

Host genetics and epigenetics are supplemented by more recently recognized ecological and systems biology approaches to biomarkers [140,141]. Given that reduced dominance of Lactobacillus species in the vaginal microbiota is linked to increased risk of persistence and progression, microbiota analysis could enhance existing triage methods. Nevertheless, technical issues are yet to be solved [17,56].

This potential of biomarker use is further improved by EVs. EVs serve as great candidates for liquid biopsy techniques since they help identify molecular cargos from a less invasive sample source. This means that any activities within both the immune system and the epithelial compartment will be mirrored through EV-related proteins and nucleic acids [100,142].

The understanding that HPV persistence results from interrelated changes in immune signaling, metabolic, and microenvironmental control points to the necessity of multi-targeted intervention tactics from a therapeutic standpoint [17,143]. Effective antiviral and anticancer responses are influenced by modulation of immune cell bioenergetics, highlighting mitochondrial pathways as potential therapeutic targets [84,144,145]. Concurrently, efforts to improve mucosal microbial balance, lower chronic inflammation, or alter epigenetic changes are being investigated as supplements to current preventative measures [146].

Precision prevention may ultimately be made possible by incorporating indicators collected from viruses, hosts, microbes, metabolism, and vesicles into composite risk models. These strategies may lessen overtreatment while guaranteeing prompt detection and treatment of women who are most at risk of advancement [147,148].

## 10. Future Directions and Outstanding Questions

There are still basic uncertainties about why some women do not recover from high-risk HPV infection, despite significant advancements in vaccination, screening, and molecular diagnoses. It will be necessary to shift from reductionist methods to integrative, longitudinal, and systems-level research to close these gaps [1,25].

Implementing longitudinal multi-omics study designs is a top goal. Cross-sectional data are widely used in current microbiome research, which limits the ability to draw causal conclusions. Determining whether dysbiosis precedes persistence or arises from infection requires prospective studies incorporating virological, immunological, genomic, transcriptomic, and microbiological measures. These methods will be essential for creating disease progression prediction models and improving precision preventative techniques [17,51,59,89].

Simultaneously, functional characterization should take precedence over mere taxonomy. While composition-based analysis has produced some very useful information, it cannot explain everything. The metabolic processes of microorganisms, their physiological impacts on immune function and epithelial integrity, and signaling pathways between hosts and bacteria should all be the subject of future research. The identification of microbial taxa alone does not adequately capture their functional and mechanistic contributions [89].

Moreover, further research regarding mitochondrial biology in HPV infection is necessary. While there is substantial evidence for a link between defective antiviral responses, cancer development, and mitochondrial impairment, there is little direct evidence in studies on cervical persistence. Analysis of mitochondrial health, interferon activity, and redox balance could help establish whether metabolic susceptibility is associated with pre-existing disease or prior to disease onset [35,41,58,62].

Similarly, research concerning extracellular vesicles also calls for standardization in methodology. Despite being potent intercellular communicators and biomarkers, variations in their isolation, characterization, and functionality pose challenges to reproducibility. It is imperative that studies involving EVs follow the set consensus guidelines for the development of clinical applications to come to fruition [103,117].

Another key route lies in incorporating new biological markers into a risk management approach. Predictive analysis that incorporates viral genotyping, host immunity, the microbiome’s structure, and other biological characteristics may improve triage tactics and minimize unnecessary treatments while retaining high sensitivity for clinically relevant conditions [130,131].

It is important to consider the international perspective on the fight against cervical cancer. An individual’s HPV infection can remain an issue among many groups owing to the disparities that exist in vaccinations, screening, and health care facilities, although there has been a paradigm shift in terms of eradication by the World Health Organization. Research on the mechanism has been crucial in accelerating the process of viral clearance [25,31,126].

Finally, new intervention approaches that aim to manipulate metabolic, inflammatory, and microbiome factors may offer fascinating avenues for exploration. Treatments aimed at improving mitochondrial function, lowering inflammation, and restoring the mucosa’s microbiota may be added to the current preventive measures. While such interventions hold great promise and are yet to undergo rigorous research and experimentation, they may offer solutions to the biological aspects of persistent infection [58,62,89].

It is apparent through the synthesis of these objectives that there is a need for interdisciplinary and integrative science. For the translation of information to address the problem of HPV persistence, as well as its subsequent consequences, it is imperative to pursue this objective [31,60,123,126].

## 11. Conclusions

Persistent infection with high-risk human papillomaviruses is the main cause of cervical carcinogenesis, while there are other underlying mechanisms at play as well. This study proposes that microbial dysbiosis, chronic inflammation, redox imbalance, mitochondrial dysfunction, and extracellular vesicle-mediated communication collectively weaken antiviral defense and promote viral persistence. However, the strength of evidence supporting these interactions varies across pathway components, and several proposed links are based on associative findings or mechanistic evidence derived from broader viral, inflammatory, and oncologic contexts rather than direct HPV-specific studies.

This framework suggests that microbial inflammation and OS may influence antiviral responses through interconnected cellular pathways. At the same time, exosomes enhance viral persistence by transporting metabolic and immunoregulatory signals. Accordingly, the proposed microbiome–mitochondria–extracellular vesicle axis should be interpreted as an integrative conceptual framework and hypothesis-generating model rather than a fully established causal pathway.

This integrative perspective highlights the importance of the coordinated dysregulation of interrelated biological systems, resulting in persistent HPV infections. This perspective helps in the development of multifunctional markers and an integrated approach for treating the infection. For better risk stratification and for the eradication of cervical cancer, this strategy must be developed further.

## Figures and Tables

**Table 1:** Cervicovaginal microbiome features and their association with HPV persistence and progression.

Microbiome Feature	Key Characteristics	Biological Effects	Impact on Host Defense	Relevance to HPV Outcomes
*Lactobacillus* species-dominated microbiome [47,48,54]	Low diversity; lactic acid production; low vaginal pH	Maintains epithelial barrier integrity; inhibits pathogen colonization	Supports mucosal homeostasis and effective antiviral responses	Associated with viral clearance and lesion regression
Anaerobic, high-diversity microbiome [50,52,53]	Enrichment of *Gardnerella*, *Prevotella*, *Sneathia*, *Atopobium*	Dysbiosis; elevated inflammatory mediators; enzymatic degradation of mucus and ECM	Disrupts immune balance and barrier function	Associated with persistence and progression
Reduced *Lactobacillus* abundance [53,55,56]	Loss of protective acidic environment	Increased epithelial permeability; reduced colonization resistance	Facilitates viral entry into basal keratinocytes	Increases risk of persistent infection
Proinflammatory microbial state [13,57,58]	Elevated cytokines and chemokines; immune cell recruitment	Chronic inflammation; OS; genomic instability	Dysregulated antiviral responses	Promotes viral persistence and disease progression
Microbial enzymatic activity [12,14,50]	Production of mucin-degrading and proteolytic enzymes	Degradation of mucus and extracellular matrix	Compromised mucosal barrier	Enhances susceptibility to infection and persistence
Temporal microbiome instability [51,59,60]	Fluctuation between CST states	Recurrent shifts in epithelial integrity and immune tone	Inconsistent antiviral protection	May contribute to episodic HPV detection
Species-specific effects [47,48,54]	*Lactobacillus crispatus* vs. *Lactobacillus iners* dominance	Differential stability and immune modulation	Variable effectiveness of mucosal defense	*Lactobacillus crispatus*: protective; *Lactobacillus iners*: less protective/transitional

**Table 2:** Intracellular molecular mechanisms linking redox imbalance, mitochondrial dysfunction, and impaired antiviral surveillance in HPV persistence.

Biological Alteration	Molecular/Cellular Consequence	Impact on Antiviral Gene Regulation	Relevance to HPV Persistence
Elevated ROS [58,62,72]	Oxidative damage to nuclear and mitochondrial DNA; disruption of electron transport chain function	Impaired MAVS-dependent IRF3 activation and reduced IFN-β transcription	Promotes genomic instability and a persistence-permissive cellular environment
Mitochondrial membrane dysfunction [74,77,80]	Disrupted MAVS platform organization and signaling complex assembly	Reduced type I interferon induction and decreased ISG expression	Weakens antiviral clearance capacity of infected keratinocytes
Altered mitochondrial metabolism [41,81,86]	Bioenergetic reprogramming and redox imbalance (OXPHOS/glycolytic shift)	Dysregulated immune cell activation and cytokine gene expression	Supports a chronic infection microenvironment
Viral interference with mitochondrial apoptosis (E6/E7-mediated) [41,66,90]	Inhibition of p53-dependent mitochondrial apoptotic pathways	Reduced clearance of infected cells and indirect suppression of antiviral signaling	Extends survival of infected, genomically unstable cells
Chronic redox imbalance [58,69,70]	Epigenetic remodeling, including DNA methylation and histone modification changes	Stable repression of interferon-stimulated genes and antigen presentation pathways	Establishes a durable persistence niche

## Data Availability

No new data were created or analyzed in this study. Data sharing is not applicable to this article.

## Abbreviations

The following abbreviations are used in this manuscript:

HPV	Human papillomavirus
ROS	Reactive oxygen species
EVs	Extracellular vesicles
OS	Oxidative stress
miRNAs	microRNAs
MAVS	Mitochondrial antiviral signaling
CIN	Cervical intraepithelial neoplasia
ISGs	Interferon-stimulated genes
CSTs	Community state types
